# Supplementary figures and images for: Innate attraction and aversion to odors in locusts
Source: PLoS One. 2023 Jul 10;18(7):e0284641. doi: 10.1371/journal.pone.0284641 (PMC10332586; doi:10.1371/journal.pone.0284641)

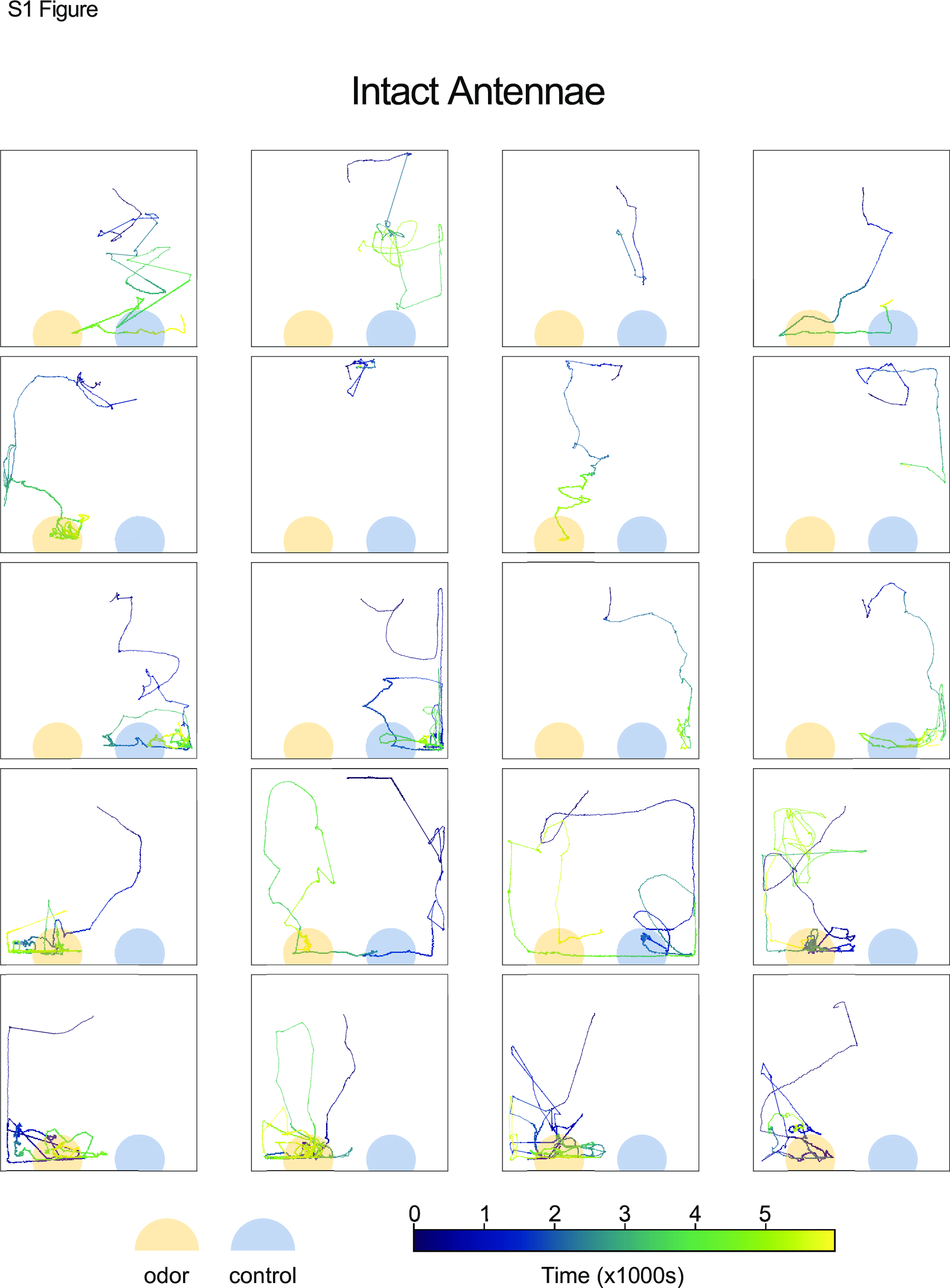

Supplement: S1 Fig — Each row shows individual animals from one experiment. Orange circle indicates the odor ROI and the blue circle the control ROI. Tracks are color-coded by time. (TIF) [file pone.0284641.s001.tif]

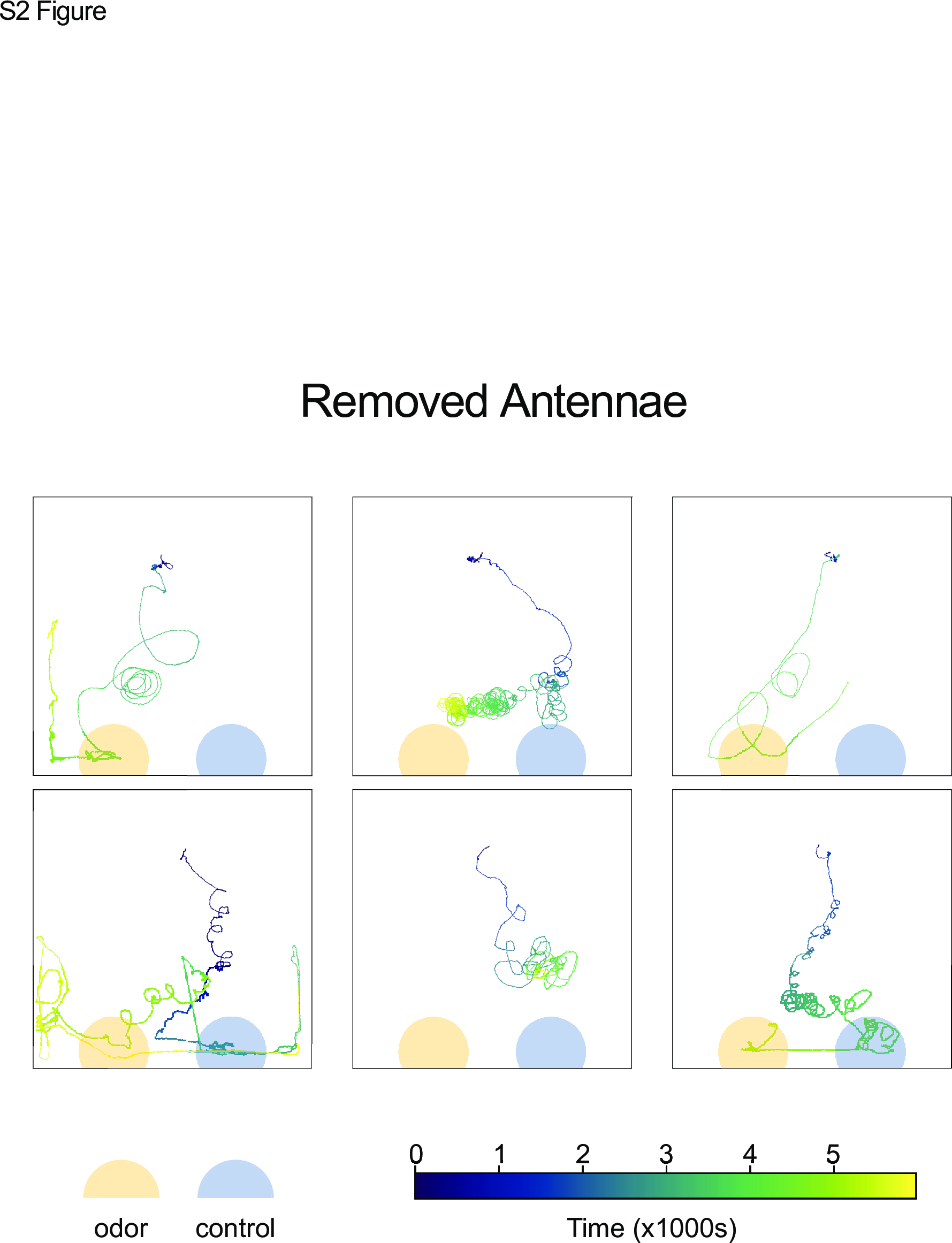

Supplement: S2 Fig — Each plot is an individual animal from a different experiment. Orange circle indicates the odor ROI and the blue circle the control ROI. Tracks are color-coded by time. (TIF) [file pone.0284641.s002.tif]
