# Supplementary material for: Innate attraction and aversion to odors in locusts
Source: PLoS One. 2023 Jul 10;18(7):e0284641. doi: 10.1371/journal.pone.0284641 (PMC10332586; doi:10.1371/journal.pone.0284641)
Supplement: Attachment — Submitted filename: Response to Reviewers.docx [file pone.0284641.s004.docx]

We are very grateful to the editors and reviewer for this very positive and helpful critique which has allowed us to dramatically improve our manuscript. In this letter we provide detailed responses to each concern. Line numbers in this letter refer to the version of the manuscript with Track Changes.

The current study has a good sample size, and I was very intrigued and impressed with the variety of observations that the authors have made. However, many details are missing and unclear in the abstract, introduction, and methods and it is not obvious what the authors have studied until the result section. Moreover, information is scattered in the method and therefore better organization in the method section is required. It was hard to understand how the tracking device works (which is the main observation tool used in the study) so a detailed explanation in the introduction/method sections is required. Finally, the authors need to mention whether the locusts are in solitarious or gregarious phases as the odor capacity changes between the two phases. Below are detailed reviews for each section.

We thank the reviewer for this very helpful and positive critique. In brief, we have now reorganized the methods section and added more information about the system we used to track the movement of the locusts. The locusts we used in this study were 1st instar, literally just crawling out of the sand in which they had hatched. At this developmental stage solitary and gregarious phenotypes are not yet distinguishable. We now clarify this below, and in the “Behavior experiments” section of the manuscript.

**Abstract**

- Need to mention whether it is gregarious or solitarious and whether lab-reared or wild-caught.

We agree and now provide this information. We used lab-reared locusts taken from our crowded colony. The animals used in this study were freshly hatched, 1^st^ instar. At this developmental stage locusts do not yet display any clear phase. Published work has shown polyphenism in this species accummulates over the entire lifecycle after hatching and becomes prominent at the 5th instar (Heifetz et al., 1996; Pener, 1991; Sword, 2003; Tanaka, 2004; Uvarov, 1966). In our own laboratory colony, we first observe changes in color in the 3rd instar, with gregarious phenotypes settling in during the 5th instar. We now clarify this under "Behavioral experiments" in line 127ff, "The phase of newly hatched locusts is indeterminate. Initially green, a characteristic of the solitary phase, our locusts typically turn brown, a characteristic of the gregarious phase, by around the 3rd instar. Phase transitions can occur at multiple time scales; individuals can switch within hours, but epigenetic changes contributing to polyphenism can develop and accumulate over several generations (Ernst et al., 2015). Our locust hatchlings are progeny of many generations of gregarious locusts."

- “*We conducted open field two-choice tests with purely olfactory stimuli. In these tests, newly hatched locusts navigated toward, and spent time near, the source of a food odor blend, crushed wheat grass*”

What do you mean by purely olfactory stimuli? Do you mean by synthetic compounds? Spent time near wheat grass more than…? What were the two choices? (e.g., control vs. wheat grass)

We thank the reviewer and have now clarified our meaning. By "purely olfactory stimuli" we meant that our test arena provided no other cue (visual, audible, mechanical) associated with the odorant. The arena had two odor ports, one on the left and one on the right. Both delivered air to the arena at equal flow rates. For each trial, one port was chosen to deliver odor; air to this port passed through a vial containing the odorant solution. Air to the other, control port, passed through a vial containing purified water. Odor and control ports were alternated between experiments. Left and right sides of the arena were equally illuminated. Therefore, the only difference between the left and right side of the arena was the presence or absence of the odorant. We have now clarified our meaning in line 21:

“We conducted open field tests in an arena designed to provide only olfactory cues to guide navigation choices. We found that newly hatched locusts navigated toward, and spent more time near, the odor of wheat grass than humidified air.”

- “*They were neither attracted nor repelled by a lower concentration of 1-hexanol, but were moderately attracted to a low concentration of hexanal. These results establish that hatchlings have a strong, innate preference for food odor blend, but the valence of the blend’s individual components may be different and may change depending on the concentration*”

What were the concentrations in “lower” and “low”?

Now we explicitly mention these concentrations in Lines 27ff: "... 1-hexanol (1% v/v) and hexanal (0.9% v/v) diluted in mineral oil relative to control presentations of unscented mineral oil. Hatchlings were neither attracted nor repelled by a lower concentration (0.1% v/v) of 1-hexanol but were moderately attracted to a low concentration (0.225% v/v) of hexanal."

- Need to mention the tracking device that was used to record the movements of locusts.

Now we have added more information about the tracking system in lines 31ff: "We quantified the behavior of the animals by tracking their positions with the Argos software toolkit.”

**Introduction**

- Expansion in the introduction section is required. Explanation of what kind of chemicals *Schistocerca americana* (or other locust species) are attracted or repelled, what method was used, and which life stage or phase was it. Moreover, the introduction does not discuss the effect of cooling and antenna amputation on odor perception in locusts, so this needs to be mentioned.

We agree and have now substantially expanded the introduction to provide the information requested by the reviewer. In lines 62ff: “A substantial literature documents chemicals attractive or repellent to locusts. Locusts are generalist foragers and consume a wide variety of plants (Capinera, 1993; Otte, 1975; Smith and Capinera, 2005). In Schistocerca gregaria, fifth instar nymphs raised on other plants are attracted to wheat grass (Njagi and Torto, 1996). Many of the volatiles present in insect food are also found in insect feces, and some of these chemicals or their derivatives may function as pheromones. For example, fifth instar nymphs of S. gregaria have been reported to show strong aggregation responses to a synthetic blend mimicking fecal volatiles including the aldehydes hexanal, octanal, nonanal, and decanal, as well as their corresponding acids (Torto et al., 1996). Two-day-old males of the oriental migratory locust (Locusta migratoria manilensis) also exhibit aggregation responses when presented an odor blend containing hexanal and other fecal volatiles (Shi et al., 2011). Among various chemicals produced by gregarious adults of Locusta migratoria 4-vinylanisole attracts, but phenylacetonitrile repels conspecifics (Guo et al., 2020).”

...

Lines 104ff: “In insects, the antennae serve as the primary olfactory organs. However, insects can also possess olfactory receptors in their mouth parts and, in some species, their ovipositors (Barton Browne, 1960; Keil, 1999; Li et al., 2020; Yadav and Borges, 2017). To evaluate the contribution of the locust antennae to odor-directed navigation, in some experiments we anesthetized the hatchlings by cooling them and then amputated their antennae. In adult locusts, cooling the antennae leads to a reversible reduction in both spontaneous and odor-elicited activity in olfactory receptor neurons (Joseph et al., 2012).”

- An explanation of the “*new software toolkit for recording and tracking* (Line 48)” is required. What is the name? How does it work? What behaviors have been recorded in past studies?

We appreciate this suggestion and have now added more information about this software toolkit, which is named Argos. Because we recently published a methods paper describing this toolkit in great detail (Ray and Stopfer, 2022), we believe it is appropriate to offer a relatively brief description of it here in the Introduction in lines 87ff: “We monitored the movements of the hatchlings within the arena by using the software toolkit Argos (Ray and Stopfer, 2022) which records videos and tracks the locations of multiple animals throughout experiments.”

We provide more information about the Argos software in the Methods section, in lines 268ff: “Locust behavior in the arena was recorded by a USB web camera (Logitech Pro Stream C922x) for 100-minute segments using the Capture utility of the Argos software toolkit (Ray and Stopfer, 2022). We then used this toolkit to analyze video segments offline, keeping only time-stamped frames that included detectable motion. We then processed these reduced videos with the Argos Tracking tool to automatically track hatchling movements. To accurately map tracks in all videos to the same coordinates, we manually marked four corners of the arena floor in each video, and then based on these points, computed the transformation matrix from video coordinates to world coordinates, and then, using this matrix, transformed video coordinates into world coordinates. Finally, because the odor and control ports were alternated between trials, we brought them into the same alignment by flipping the coordinates left-right as necessary. Afterwards, the timings of hatchling movements were reconstructed from the timestamp associated with each video frame.”

- “*We found that even without any prior exposure to food, hatchlings could navigate to the source of a complex food odor blend, wheat grass juice. Notably, while attracted to the complex food blend, hatchlings were repelled by even low concentrations of the major component of the blend, 1-hexanol, presented alone. We also found the hatchlings were attracted by low concentrations of hexanal, which is both a food blend component and an aggregation pheromone but avoided it at higher concentrations. Thus, our results show that naïve hatchlings are innately attracted by food odor, that they can navigate to its source, and that individual monomolecular components of an attractive food odor can change valence depending on concentration*.”

These sentences should be in the discussion but not in the introduction. Rather than writing about the results from the study, questions asked in the study, prediction and reasoning behind the prediction should be included.

We have now removed these sentences from the introduction.

**Method**

- Lines 99-108 in the subsection *Behavior experiments* should be placed at the beginning of the method as it explains the information on insect husbandry. It also needs to mention where the insects were collected (or purchased) and the sample size for each experiment. Also, in what conditions (temperature, humidity, light cycle) was the wheat grown? Where were the wheat seeds purchased or collected from?

We have now moved this section to the beginning of the methods section and have added information about our locust colony and wheatgrass. We have also added the number of animals used in each experiment in addition to the number of tracks that were considered for analysis (we analysed tracks only from animals that visited at least one stimulus port).

- Was the arena placed outside or inside that lab? Where (geolocation, name of the institute) was it conducted?

The arena was placed inside a room in our lab at Porter Neuroscience Research Centre, National Institutes of Health. We have now added this information to the methods section.

- Need to clearly state what behavioural signals that you have used to observe attraction/repulsion between control vs. food smells, with or without antenna, with or without cooling

We have now added this information to the “Behavior experiments” subsection.

- I think it would be better if you combine the subsections *Behavior arena*, *Odor delivery* and *Odor intensity map in arena* because they all explain the arena setups and refer to the same figure

We have now merged these subsections.

- To what concentrations the odorants were diluted? Need to mention in the method section too (not just in the result section)

We now added this information in the method section under the “*Odorants”* subsection.

- What chemicals were used to test olfactory capability in locusts with or without antennae or with or without cooling?

We tested freshly extracted grass juice to do these tests. We have now made this explicit in the “*Odorants”* subsection.

- Information on survival probability (Figure 2D) is missing. The method used to measure survival rate (Kaplan-Meier method) was first mentioned in the result section.

We appreciate this concern and note that the Kaplan-Meier method is discussed in the *Data analysis* subsection of the Methods. To clarify this, we have now added a Figure panel identifier in this Methods paragraph, lines 300ff:

“To compare attraction toward the test odor port and the control port over time we used the lifelines module in Python for survival analysis with Kaplan-Meier fit. The Kaplan-Meier curve is a step function indicating the probability of “survival,” i.e. the probability that an event of interest has not yet occurred. In the experiments comparing attraction towards grass juice odor and control, we computed, for each hatchling, the interval between first detecting the hatchling in the arena and its first entrance to the ROI around the odor port. We used these intervals to make a survival plot, with the entry into the ROI as the event of interest (orange line in Fig 2d). Animals which did not enter the ROI within the experiment duration of 100 minutes were “censored.” Similarly, the first entry into the ROI around the control port was the event of interest for control, and the time interval from first detecting the hatchling until this event was used in the Kaplan-Meier fit (blue line in Fig 2d). The log-rank test was used to compare these curves. Note that here we are comparing the first entry into the odor ROI with that into the control ROI, and an animal entering the two ROIs is not mutually exclusive; if a hatchling visited both ROIs, then it is included in both curves with the corresponding time intervals. For example, an animal could first enter the odor ROI at time t1, and then move into the control ROI at time t2. Then t1 would be included in the survival plot for odor, and t2 would be included in the survival plot for control.”

- Abbreviations in figure legends must be in the full name (e.g., PID in Figure 1, ROI in Figure 2). What do circles with different sizes indicate in Figure 1b & c?

We have now expanded all the abbreviations in the figure legends. Circles of different size indicate relative odor intensity. We have now made this explicit in the legend: “Circle diameter indicates odor intensity.”

**Result**

- Please remove all “(see Materials and Methods)”

We removed all such references in the manuscript.

- What are circular paths?

To clarify this point, we’ve rewritten this sentence to say: “We noted that these antennectomized hatchlings, unlike intact ones, tended to walk in looping arcs” (line 261ff). We understand that this result is probably not clear from figure 3a because many paths overlap. Therefore, we have now added a supplementary figures “S1” and “S2” showing sample tracks of normal locusts in the test with grass juice, and individual examples of tracks of antennectomized locusts, respectively.

- Sentences with references have to be moved into the introduction or discussion section (e.g., Line 190 “…earlier experiments’; Line 201-205 From “The major monomolecular volatile…” until “antennal lobes (Stopfer et al, 2003)”.

We have now moved all sentences with references to either the introduction or the discussion section.

- The subheading “*Attraction to food odor is mediated by antennae*” is contradicting as the locusts are still attracted towards the food odor without antennae. It would be clearer if you could compare attraction to odor source between locusts with or without antennae (also for the cooling effect analysis; compare attraction between with or without cooling)

We regret our unclear wording. As we demonstrate in Figure 3, the animals without antennae did not show any differences in their movement towards the food-odor blend port compared to the control port. Thus, the normal preference for food odor is lost when the antennae are removed. In contrast, control animals which were cooled and then revived to room temperature, leaving the antennae intact, showed the normal preference for food odor. We have now revised our text to make this clear.

Line 364ff: “We found hatchlings lacking antennae showed no preference for the food odor port compared to the control port (Figure 3a-c; two-tailed paired t-test, for stay time n = 35, p = 0.44, PI = -0.10, and for number of ROI entries n=35, p= 0.57, PI=0.07, showing no significant difference).“

**Discussion**

- Line 241 “…, *a plant readily consumed by locusts of all ages*”

Reference?

We have now added a reference for this (now line 452ff): “..., a plant readily consumed by locusts of all ages (Chapman and Sword, 1994; also, our own unpublished observations).”

- Line 241 “*We also found that hatchlings require their antennae to navigate towards the odor source*”

Again, this is contradicting as hatchlings without antennae are attracted towards the smells. Also, it would be good to discuss past studies that have explored odor perception in grasshoppers after antenna amputation and cooling

As we note above, we regret our confusing writing – our observations, in fact, demonstrated that hatchlings without antenna are not attracted toward smells. We have now revised our text to clarify our meaning.

We agree that it would be helpful to discuss past studies relevant to cooled or cut antennae, and have now added the following paragraph (line 458ff):

“Insects require their antennae to navigate toward odor sources, as demonstrated by experiments employing antenna amputation in honeybees (Karl von Frisch, 1927) and cockroaches (Glaser, 1927). Experiments with cockroaches suggested that the palp olfactory system may be engaged primarily for odorants at very short distances, whereas longer distances require the antennal olfactory system (Glaser, 1927). Antennectomy has been shown to reduce overall locomotion in 5th instar locusts and to impair their tendencies to walk upwind and toward odors, although palp-mediated olfaction may, in this developmental stage, enable some odor-guided movement (Kennedy and Moorhouse, 1969). Consistent with this earlier work, our experiments with antennectomized locust hatchlings also revealed reduced and atypical locomotion; however, we found no evidence for palp-mediated olfactory guidance. To anesthetize hatchlings, we briefly cooled them. Cooling has also been shown to reversibly reduce spontaneous and odor-elicited activity in olfactory receptor neurons and to increase behavioral sensitivity to odors in insects (Joseph et al., 2012; Martin et al., 2011; Riveron et al., 2009). In this study we did not specifically investigate cooling-induced changes in olfactory sensitivity, but we observed that in animals with intact antennae that had been cooled, olfactory capabilities recovered fully upon warming to room temperature.”

- Odor perception in mice and *Drosophila* are discussed but it would be better to focus on locusts which are well-studied in the past. For example, response to the wheatgrass smells and to green leaf volatile was observed in locust hatchlings in this study, but how about in adults of *S. americana*? Or in other locust species (like *Locusta migratoria* and *Schistocerca gregaria*)? The idea Line 255 “*The diet of mother mice has been shown to influence olfactory neurodevelopment and odor preference in the newborn (Todrank et al., 2011). This possibility could be tested in locusts by manipulating the mother’s diet or the volatiles present in egg pods*” is very interesting so expansion of this idea would be good for discussion

We agree. As noted above, we have now added a paragraph that discusses responses of adult locusts of several species. We have also added the following to the paragraph mentioned by the reviewer (lines 479ff): “One could, for example, rear a generation of locusts on a synthetic diet (Simpson et al., 1988) to avoid the deposition of plant material along with the egg pods. Afterwards, offering hatchlings from such egg pods a choice of grass juice volatiles or odorants present in the synthetic food could reveal whether the presence of chemicals in the mother’s diet is responsible for the preference of the hatchlings for these chemicals.”

- “Low” or “lower” concentration is very vague and needs to be clear about what concentrations were used in this study and what concentrations (with which solvents) have been tested in past studies.

We agree and now provide this information in the text. We have also added references to prior studies. See lines 535ff: “Animals may have different behavioral thresholds for navigation and consummatory behaviors. For example, fifth instar locusts will display a palp opening response (POR) when many types of odorants in filter paper are presented, including hexanol and hexanal at concentrations avoided in our locomotion experiments (Wang et al., 2019). Olfactory neurons in locusts have been shown to respond to wide ranges of concentrations of odorants (for example, the headspace over 0.1% to 100% of 1-hexanol dissolved in mineral oil; Kim et al., 2023; Stopfer et al., 2003). To further elucidate the neural basis of innate olfactory behaviors, it will be important to investigate a wider range of odor concentrations.”

We note that we have also removed a paragraph in the discussion section about the possibility of labeled line processing in mouse and Drosophila as our study does not directly address this issue.

Additionally, we have reformatted our manuscript to meet PLOS ONE guidelines.
